# Subdural Effusions with Hydrocephalus after Severe Head Injury: Successful Treatment with Ventriculoperitoneal Shunt Placement: Report of 3 Adult Cases

**DOI:** 10.1155/2010/743784

**Published:** 2010-12-12

**Authors:** N. Tzerakis, G. Orphanides, E. Antoniou, P. J. Sioutos, S. Lafazanos, A. Seretis

**Affiliations:** ^1^Department of Neurosurgery, Athens General Hospital, “G. Gennimatas,” Mesogeion 154, 11527 Athens, Greece; ^2^8 Shirlaw close, Westerhope, Newcastle upon Tyne NE5 4DG, UK

## Abstract

*Background*. Subdural collections of cerebrospinal fluid (CSF) with associated hydrocephalus have been described by several different and sometimes inaccurate terms. It has been proposed that a subdural effusion with hydrocephalus (SDEH) can be treated effectively with a ventriculoperitoneal shunt (V-P shunt). In this study, we present our experience treating patients with SDEH without directly treating the subdural collection. *Methods*. We treated three patients with subdural effusions and hydrocephalus as a result of a head injury. All the patients were treated with a V-P shunt despite the fact that there was an extra-axial CSF collection with midline shift. *Results*. In all of the patients, the subdural effusions subsided and the ventricular dilatation improved in the postoperative period. The final clinical outcome remains difficult to predict and depends not only on the successful CSF diversion but also on the primary and secondary brain insult. *Conclusion*. Subdural effusions with hydrocephalus can be safely and effectively treated with V-P shunting, without directly treating the subdural effusion which subsides along with the treatment of hydrocephalus. However, it is extremely important to make an accurate diagnosis of an SDEH and differentiate this condition from other subdural collections which require different management.

## 1. Introduction

External hydrocephalus is a well-established entity in infants which is benign and usually resolves without shunting [1, 2]. The term “External Hydrocephalus” has also been used to describe the presence of extra ventricular cerebrospinal fluid (CSF) collections accompanied by hydrocephalus, particularly in cases of adults suffering from aneurysmal subarachnoid hemorrhage and severe head injuries [3–6]. Several other terms have been used to describe this entity [7] which has lead to confusion about this disease. 

However, the fact that this form of hydrocephalus does not have a benign course and needs in many cases surgical management [3, 6–9] demonstrates the need for a different term other than “external hydrocephalus.” The term subdural effusion with hydrocephalus (SDEH) has been used in the literature previously [6, 8] and describes more accurately the nature and the severity of this condition, thereby differentiating it from the benign subdural collections of infancy and subdural hygromas. 

 A subdural peritoneal (S-P) shunt or single burr hole drainage are the preferred methods of treating subdural hygromas [10]. This opinion has been challenged and in a retrospective study of 1,601 patients with brain injury, conservative management has been proposed for delayed evolution of posttraumatic subdural hygroma [11] because of modest improvement after operation. However, if an SDEH is treated as a simple subdural hygroma, after the S-P shunt placement ventricular dilatation supervenes and the patient will need a ventriculoperitoneal (V-P) shunt thereafter. It is extremely important to differentiate SDEH from other subdural effusions such as hygromas and chronic subdural hematomas because the V-P shunt placement in cases of subdural collections without hydrocephalus will increase the collection and may lead to neurological deterioration. On the other hand, if an SDEH is regarded as a subdural hygroma, the treatment of the hydrocephalus is delayed, which may lead to a permanent neurological deficit. 

In addition, the management of subdural effusions may require multiple unsuccessful surgical procedures (burr hole drainage of the subdural collection or S-P shunts). These procedures have only a temporary effect in cases of SDEH, since the real cause of this condition is the hydrocephalus and the communication between the ventricles and the subdural space which allows the CSF to be diverted outside the ventricles. Any attempt to treat the subdural collection directly, in cases of SDEH, before the permanent management of the hydrocephalus increases significantly the risk of developing a central nervous system (CNS) infection with subsequent further delay in V-P shunt implantation. To follow, there are three illustrative cases, demonstrating patients who successfully underwent a V-P shunt placement (OSV II Smart ValveT System, Integra Neurosciences Implants S.A.) for the treatment of a subdural collection with hydrocephalus, following a head injury.

## 2. Case Presentations

**Case 1**

The first patient was a 67-year-old man who was transferred to the Accident and Emergency Department disorientated, with a Glasgow Coma Score (GCS) of 10/15 (global aphasia, not obeying commands) following a moderate head injury, which he sustained in a road traffic accident.

**Figure 1:**
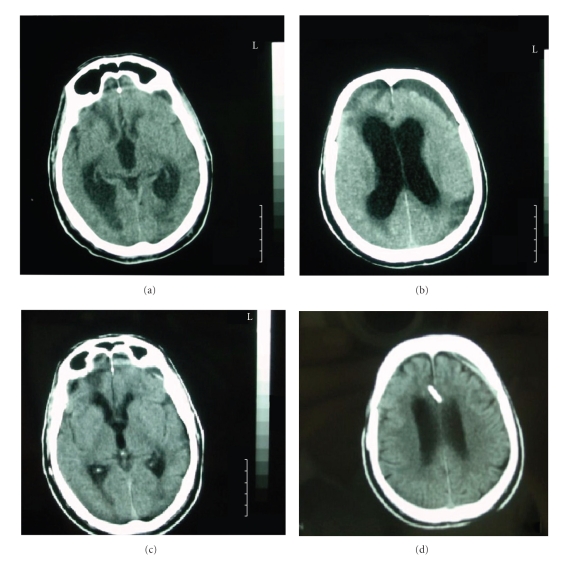
CT scan showing the left frontal subdural effusion associated with hydrocephalus (a) and (b) treated with V-P shunt.

A computerized tomography (CT) scan revealed posttraumatic subarachnoid hemorrhage and right frontal petechial contusions which did not require evacuation. The patient was completely aphasic for the first 72 hours after the injury. A subsequent CT scan revealed bilateral subdural hypointense collections although the patient remained neurologically stable. However, on day 4 after the injury he deteriorated gradually and a new CT scan revealed enlargement of the left subdural collection with dilatation of the ventricles (Figures 1(a) and 1(b)). A lumbar puncture was performed, after which the patient initially improved but unfortunately he deteriorated again. The decision was made to treat this condition as an SDEH, and as a result, a V-P shunt was inserted. On the day that the operation was performed, the patient had deteriorated to a GCS of 8/15.

In the immediate postoperative period, he improved gradually, and 7 days after the operation, he regained speech and began to mobilise. A followup CT scan (Figure 1(c) and 1(d)) three months after the operation showed normalization of the ventricles, and the subdural collection was almost absent. Furthermore, the patient's symptoms were completely resolved, and the outcome was classified as “good recovery” using the Glasgow Outcome Scale (GOS = 5).

**Case 2**

The second case was a 47-year-old man who sustained a severe head injury following a road traffic accident. He was intubated and ventilated before being transferred to the Accident and Emergency Department. His initial GCS was 5/15. A head CT scan demonstrated a subdural hematoma in the left frontotemporoparietal region, hemorrhagic contusions in the same area with mass effect, and evidence of a posttraumatic subarachnoid hemorrhage.

He was immediately transferred to the theatre where he underwent a left frontotemporoparietal craniectomy because of the cerebral edema and removal of the subdural hematoma. Before the operation, the left pupil was fixed and dilated, he had a right hemiplegia and he was extending to pain.

**Figure 2:**
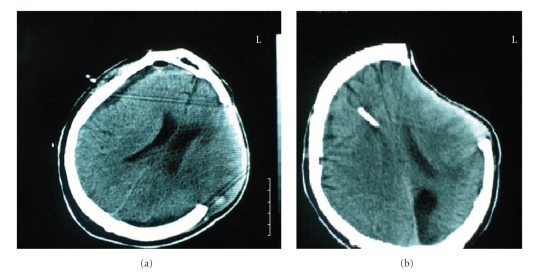
Postcraniectomy SDEH with bulging of the brain through the craniectomy defect (a) which is resolved after the treatment of hydrocephalus with a V-P shunt.

A followup CT scan showed complete removal of the hematoma, but the subsequent scans revealed a subdural CSF collection with ventricular dilatation (Figure 2(a)). Due to brain herniation he developed a left posterior cerebral artery (PCA) infarct. Initially, attempts were made to remove the subdural collection with a burr hole, but this procedure was unsuccessful as the collection recurred.

The condition was treated as an SDEH and a V-P shunt was inserted. The subdural effusion disappeared, and the hydrocephalus was successfully treated (Figure 2(b)). He underwent a cranioplasty 6 months after the V-P shunt was inserted. However, he remains severely disabled, obeying simple commands, and opening his eyes spontaneously (GOS: 3).

**Case 3**

The last case was a 69-year-old man who was intubated and ventilated before transfering to the Accident and Emergency Department from a district hospital, following a road traffic accident. His initial GCS at the scene was 5/15.

A CT scan revealed a subdural hematoma in the left frontotemporoparietal region, a large hemorrhagic contusion in the left temporal lobe with mass effect, and a posttraumatic subarachnoid hemorrhage. He underwent a left frontotemporoparietal craniectomy and removal of the subdural hematoma.

**Figure 3:**
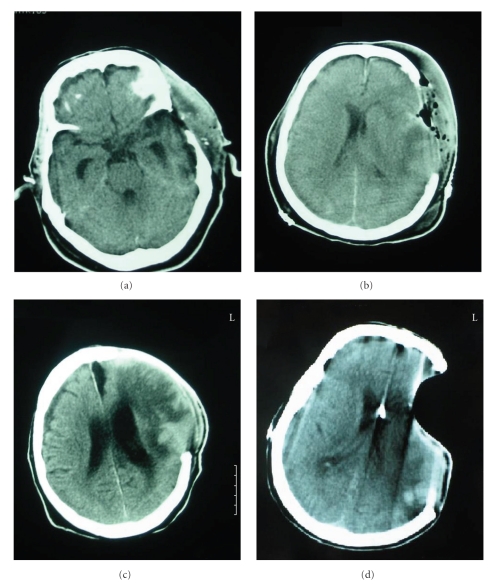
Satisfactory removal of an acute subdural hematoma (a), but the followup scan revealed ventricular dilation with a right subdural effusion and widening of the interhemispheric fissure (b) and (c), which were successfully treated with a V-P shunt.

The patient remained intubated and the first followup CT scan showed that the hematoma had been successfully removed (Figure 3(a)). 24 days later, a CT scan revealed ventricular dilation with a right subdural effusion and widening of the interhemispheric fissure (Figures 3(b) and 3(c)).

Serial lumbar punctures were performed because a V-P shunt could not be inserted due to serious infections. After a long period of antibiotic treatment, the patient underwent a V-P shunt placement. The post-op CT scan revealed normalization of the ventricular size and absence of the right frontal and interhemispheric subdural effusion (Figure 3(d)). The patient still remains severely disabled (GOS: 3).

## 3. Discussion

Subdural effusions with hydrocephalus (SDEH) in adults have been described after aneurysm rupture and subarachnoid hemorrhage, [3, 4, 6, 7] after neurosurgical procedures [3, 4, 6, 8] and severe head injuries [3, 4]. 

### 3.1. Pathophysiology

The pathophysiological mechanisms of this disorder include the free communication of the ventricles, and the subdural space due to the rupture of some part of the arachnoid membrane, particularly basal cisterns or lamina terminalis tear, which then allows fluid to flow into this compartment. The SDEH occurs when the abnormal CSF circulation is combined with communication between the subdural space and the ventricles. The CSF is diverted to the subdural space because the convexity of the brain has less resistance compared to the ependyma of the ventricles and the formation of the subdural CSF collection requires less pressure than the ventricular enlargement [8]. In addition to the free communication between the ventricles and the subdural space, the dysfunction in CSF absorption at the level of the arachnoid granulations is necessary for the development of a CSF subdural effusion with hydrocephalus. 

Severe head injuries are associated with hydrocephalus because of abnormal CSF circulation due to posttraumatic subarachnoid haemorrhage, arachnoid tear, cranial surgery, and particularly craniectomy [12–17]. According to Kilincer and Hamamcioglu [13], head trauma itself can cause subdural effusion due to subarachnoid haemorrhage, ruptured arachnoid tear, and gradual shrinkage of the swollen brain. The authors experienced similar difficulty with our cases in treating persistent subdural effusions, and they hypothesized that they might be the result of a “resistance gradient” between the two hemispheres caused by a unilateral large craniectomy.

 In a large series of 108 consecutive decompressive craniectomies [15], the incidence of posttraumatic hydrocephalus was 9.3%. In the same series, 21.3% of the patients had posttraumatic subdural effusion. In this study, the coexistence of the two pathologies in the form of SDEH has not been addressed. The primary problem in SDEH is the hydrocephalus, and we do agree with the opinion of Yang et al. for surgical intervention “as soon as possible after the diagnosis of hydrocephalus and the exclusion of contraindications”. 

Aarabi et al. [12] studied the dynamics of subdural hygromas following decompressing craniectomy (DC). In their series of 68 patients who underwent DC, there were 39 patients who developed hygromas and 29 who did not. The authors concluded that although hygromas are commonly (57%) developed after craniectomies, they rarely require surgical intervention since they gradually disappear. However, the hydrocephalus which was developed in patients with or without hygroma was treated with CSF diversion.

Yang et al. [16] proposed another interesting explanation of the subdural effusions after treating this complication in a patient with a decompressive craniectomy. They noticed that the patient was still treated with dehydration despite the fact that the oedema has subsided. Simply rehydrating the patient resolved the collection and the symptoms.

### 3.2. Difference between Subdural Effusions with Hydrocephalus (SDEH) and a Subdural Hygroma

The pathophysiological mechanisms that have been proposed for the formation of the traumatic subdural hygroma involve the arachnoid tearing which acts as a one-way valve between the subarachnoid and the subdural space and is usually caused by mild or moderate trauma [8]. There is also a theory that serum fluid leaks from fenestrations of small vessels on subdural neomembranes and concomitant enlargement of the subdural hygromas [10].

### 3.3. Methods of Diagnosis

Obviously, it is very important to differentiate SDEH from other subdural collections, for example, chronic subdural hematomas (CSDHs) and subdural hygromas because a V-P shunt is the treatment of choice in SDEH, but in the other cases, it will cause an enlargement of the subdural collection and a deterioration of the mass effect. The CT scan reveals, in most of the cases, dilatation of the lateral ventricles and periventricular lucency when the CSF accumulates in the subdural space [6]. However, the subdural hygroma cannot be differentiated radiographically from an SDEH before the stage of ventriculomegaly [8]. Another point is that the composition of the subdural collection can be evaluated from the signal intensity [3], especially with brain magnetic resonance imaging (MRI). This is very helpful for the diagnosis of a CSDH. The subdural hygroma contains xanthochromic fluid, and the protein content is often higher than that of CSF [10]. In addition, the subdural hygroma shows meningeal enhancement on gadolinium-diethylenetriamine pentaacetic acid- (Gd-DTPA-) enhanced MRI. Radiological evaluation of SDEH reveals preservation of the ipsilateral sulci and basal cisterns. On the other hand, CSDH and subdural hygromas produce compression of the subarachnoid spaces on the same side as the fluid collection [10]. Another radiological examination, proposed as helpful to differentiate SDEH, is CT cisternography [6] which may show whether the subdural space communicates with the ventricles; however, it has been proved inadequate test in the diagnosis of normal pressure hydrocephalus (NPH) [18, 19], and there is no indication that it will be useful investigating complex cases of SDEH. Simple lumbar punctures with removal of 20 cc of CSF may be useful but carry an obvious risk particularly with regards to subdural hygromas or CSDH. There might be a scope of using acetazolamide in the diagnosis of SDEH if the clinical condition of the patient allows for a delay in the V-P shunt implantation [20]. It has already been used for the treatment of external hydrocephalus in infants [21]. This center has used acetazolamide in some cases of extra-axial collections of CSF after craniectomies with significant but temporary results. 

The presence of an extra-axial collection with mass effect makes the decision to treat an SDEH with a V-P shunt difficult and many surgeons would argue that it is better to wait until the subdural collection has been absorbed and the hydrocephalus is established [6]. However, this practice is not without risk because shunting at a later stage might not reverse a neurological deficit. Also, dealing with the subdural collection first with a simple burr hole evacuation of the subdural effusion is not without risks; the cause of the SDEH remains untreated and the patient might develop CSF leak and subsequently infection which will delay further the implantation of the V-P shunt. After diagnosis of SDEH has been made, we would advise to treat this condition with a V-P or an L-P shunt [6]. Another approach to diagnose these difficult cases of ventriculomegaly is to use CSF dynamics [22] calculating the resistance for CSF absorption. Both of these tests require CSF removal with a lumbar puncture which carries a potential risk in the presence of a subdural hematoma or hygroma. Also, a negative tap test does not exclude the diagnosis of hydrocephalus [23, 24]. In posttraumatic patients, the ventriculomegaly may be associated with altered CSF dynamics [25] which might produce dubious results in infusion studies.

 Alternative to the tests requiring a lumbar puncture is the continuous monitoring of intracranial pressure (ICP). This has been proposed by Poca et al. as a helpful diagnostic method, particularly in complex cases, and it seems that high mean ICP and plateau waves are good prognostic factors for satisfactory outcome after shunt insertion [23, 25]. Recently, Huh et al. [7] suggested measuring the subdural pressure using a manometer intraoperatively, before opening the dura mater, in patients with subdural collections and ventriculomegaly. They found that four patients with subdural pressures above 15 cm H_2_O and a pediatric patient (2 years old) with a subdural pressure of 12 cm H_2_O eventually required a shunt operation. Both methods will be extremely helpful in managing patients with SDEH because there is no need for a lumbar puncture which carries the risk of increasing the size of the subdural collection in cases of a misdiagnosed subdural hygroma.

### 3.4. Treatment

In patients with SDEH after a severe head injury or intracranial aneurysm clipping, the placement of a V-P shunt may be sufficient to treat both the subdural effusion and the hydrocephalus and subsequently improve the clinical symptoms. The V-P shunt placement drains the hydrocephalus which is the cause of this entity, and as a result it prevents the CSF diversion to the subdural space. 

Yang et al. [15] have suggested that duraplasty could prevent the alteration in CSF dynamics after a craniectomy. In their series, the duraplasty decreased the incidence of subdural effusion. Although the authors did not mention any difference in the incidence of hydrocephalus, duraplasty is a measure to avoid the disturbance in the CSF circulation and facilitate the future cranioplasty protecting the brain tissue during dissection. According to Kilincer and Hamamcioglu [13], acknowledging the “resistance gradient” caused by a large unilateral craniectomy is important to decrease the complication rates applying modifications in the surgical technique. Duraplasty is a simple technique which might prevent subdural effusions and decrease complication rates after craniectomies. Another simple measure to correct the pressure gradient is bandaging the head after the peak time of cerebral swelling to avoid brain herniation [16].

Early cranioplasty has also been proposed [15–17, 26–29] for correction of CSF hydrodynamics after decompressive craniectomy particularly in cases of the “syndrome of the trephine”. Delayed cranioplasty was correlated with persistent hydrocephalus in a retrospective study of ten patients who underwent decompressive hemicraniectomy for ischemic or hemorrhagic stroke [17], and based on this observation, the authors suggested that early cranioplasty might promote spontaneous improvement of hydrocephalus. Although this is a different patient group from our cases—there were no trauma patients in the Waziri et al. study—this observation is important, and we would agree that early cranioplasty is appropriate providing that there are no contraindications. The two patients in our study who had a craniectomy before developing SDEH (Cases 2 and 3) were not medically fit for an early cranioplasty.

When treating a patient with a subdural effusion, it is important to consider whether there is accompanying hydrocephalus. Apart from the radiological evaluation, clinical tests including measurement of the subdural pressure are recommended providing that there is a suspicion of an SDEH. Although our patients responded to V-P shunt placement, in persistent cases, there might be an indication to proceed to an S-P shunt either in parallel or connected to the valve of the V-P shunt.
